# HDAC6 Promotes Host Defense Against Chlamydial Lung Infections by Regulating M2-Th2 Responses

**DOI:** 10.3390/ijms27073009

**Published:** 2026-03-26

**Authors:** Jinxi Yu, Shuaini Yang, Xiaoyu Zha, Yuqing Tuo, Ruoyuan Sun, Hong Zhang, Lu Tan, Hong Bai

**Affiliations:** Key Laboratory of Immune Microenvironment and Disease of the Ministry of Education, Tianjin Institute of Immunology, Department of Immunology, Tianjin Medical University, Tianjin 300070, China; yujinxi1107@tmu.edu.cn (J.Y.); nier1998@163.com (S.Y.); zhaxiaoyu@tmu.edu.cn (X.Z.); tuoyuqing2021@163.com (Y.T.); sunry0609@163.com (R.S.); zhanghong0621@tmu.edu.cn (H.Z.); tanlu@tmu.edu.cn (L.T.)

**Keywords:** HDAC6, *Chlamydia muridarum*, respiratory infection, macrophage polarization, T cell differentiation, Th2-M2 responses

## Abstract

Histone deacetylase 6 (HDAC6), a member of the class IIb HDAC family, plays a crucial role in epigenetic regulation and cytoskeletal dynamics, while participating in host anti-infective immune responses. However, its precise functions and mechanisms during *Chlamydia muridarum* (*C. muridarum*) infection remain incompletely defined. Our study demonstrated that *C. muridarum* respiratory infection upregulates HDAC6 expression at the infection site and in immune organs. Comparative analysis of wild-type (WT) and HDAC6-deficient (HDAC6^−/−^) mice in this infection model revealed that HDAC6 deficiency exacerbates disease progression, including significant weight loss, severe pulmonary inflammation, and impaired *C. muridarum* clearance. Relative to WT mice, HDAC6^−/−^ mice exhibited elevated Signal Transducer and Activator of Transcription 6 (*Stat6*) and GATA Binding Protein 3 (*Gata3*) mRNA expression, enhanced pathological Th2 responses with increased IL-4 secretion, and no significant differences in protective Th1 or Th17 responses following *C. muridarum* infection. Concurrently, these mice displayed enhanced M2 macrophage polarization, as evidenced by upregulated CD206 and Arg-1 expression, whereas M1 marker expression remained unchanged. The vitro studies confirmed that HDAC6^−/−^ bone marrow-derived macrophages (BMDMs) promote M2 polarization, characterized by increased Arg-1, IL-10, and TGF-β production, and further co-culture experiments showed that *C. muridarum* -stimulated HDAC6^−/−^ BMDMs drive Th2 differentiation. These findings elucidate the critical role of HDAC6 in regulating Th2-M2 immune responses during *C. muridarum* respiratory infection and suggest targeted modulation of HDAC6 as a novel therapeutic strategy for chlamydial respiratory infection.

## 1. Introduction

*Chlamydia trachomatis* (*C. trachomatis*), an obligate intracellular Gram-negative bacterium, infects mucosal surfaces and is a leading cause of trachoma and urogenital tract infections, posing a significant global health burden [1,2]. While *C. trachomatis* primarily targets ocular and urogenital epithelia, it can also cause respiratory diseases such as pneumonia in infants [3]. During intracellular infection, *C. trachomatis* actively suppresses host cell apoptosis to preserve its replicative niche and complete its biphasic developmental cycle [4]. At the end of this cycle, host cell lysis facilitates the release of infectious progeny, contributing to tissue inflammation, pathogen dissemination, and immunopathological effects [5,6]. Notably, mouse models infected with *Chlamydia muridarum* (*C. muridarum*), a murine pneumonitis strain, are widely used to study chlamydial pathogenesis. This model accurately mirrors the immune responses observed in humans during natural *C. trachomatis* infection, making it a reliable tool for investigating the mechanisms of chlamydial respiratory infection. Therefore, the present study employed the *C. muridarum* respiratory infection model to explore immune regulatory mechanisms underlying chlamydial infection.

Histone deacetylase 6 (HDAC6), a unique member of the HDAC family, has garnered significant attention due to its cytoplasmic localization and involvement in the deacetylation of non-histone substrates. HDAC6 plays a pivotal role in pathogen–cell fusion, entry, replication, assembly, and release, and is closely associated with processes such as cytoskeletal reorganization, cell motility, and protein degradation [7]. Research has shown that HDAC6 exhibits dual roles in infection and immunity: on one hand, it modulates the expression of inflammation-related genes and mediators to combat infections and suppress excessive inflammatory responses; on the other hand, it regulates cellular autophagy to facilitate pathogen clearance [8,9,10]. For instance, bacterial or viral infections activate the TLR signaling pathway, leading to the upregulation of HDAC6 expression and subsequent deacetylation of α-tubulin, which inhibits pathogen dissemination [11,12,13]. In *Clostridium difficile toxin A* infection, HDAC6 inhibition reduces the production of pro-inflammatory factors and mitigates mucosal damage [14]. Similarly, in LPS-induced acute lung injury, HDAC6 inhibition blocks IκB phosphorylation and NF-κB activation, thereby reducing inflammasome activity [15]. However, in infections with *Salmonella enterica*, *Salmonella typhi*, and *Listeria monocytogenes*, HDAC6-deficient mice exhibit significantly higher bacterial loads in myeloid cells compared to wild-type mice, indicating context-dependent roles of HDAC6 in different infection models [13,16].

The polarization of T cells and macrophages is a central mechanism in host defense against pathogens and the maintenance of immune homeostasis. Th1 cells and M1 macrophages mediate pathogen clearance through the secretion of pro-inflammatory cytokines such as IFN-γ and TNF-α, while Th2 cells and M2 macrophages contribute to tissue repair and immune suppression via anti-inflammatory factors like IL-4 and IL-10. However, polarization imbalance can lead to immunopathological damage or persistent infections. Studies have shown that HDAC6 plays a critical role in regulating T cell and macrophage polarization through epigenetic modifications and signaling pathways (e.g., STAT3/STAT6, GATA3, PPARγ) [17,18]. For example, HDAC6 deficiency significantly enhances STAT6 acetylation, promoting Th2 cell differentiation and M2 macrophage polarization, thereby exacerbating immune suppression in allergic or parasitic infections [19,20]. In bacterial or viral infections and tumor models, HDAC6 deficiency may impair host pathogen clearance by suppressing M1 or promoting M2 macrophage polarization [21,22]. Furthermore, the interaction between T cells and macrophages (e.g., the Th2-M2 axis) amplifies polarization imbalances. For instance, Th2 cells secrete IL-4/IL-13 to drive M2 macrophage polarization, while M2 macrophages enhance Th2 responses via CCL17/CCL22, creating an immunosuppressive microenvironment [23,24].

Although existing studies have partially elucidated the mechanisms by which HDAC6 regulates immune cell polarization, its specific molecular pathways in modulating T cell–macrophage interactions during *Chlamydia* infections remain unclear. This study is the first to focus on the role of HDAC6 in regulating T cell and macrophage polarization and its impact on infection-induced immune responses, aiming to provide new perspectives on host–pathogen interactions and lay the groundwork for immune homeostasis-based therapeutic strategies. By uncovering the immune regulatory mechanisms of HDAC6 in *Chlamydia* infections, this research holds promise for identifying novel immunotherapeutic targets, developing effective vaccines, and alleviating inflammatory damage.

## 2. Results

### 2.1. HDAC6 Upregulation Is Induced by C. muridarum Respiratory Infection

**Figure 1:**
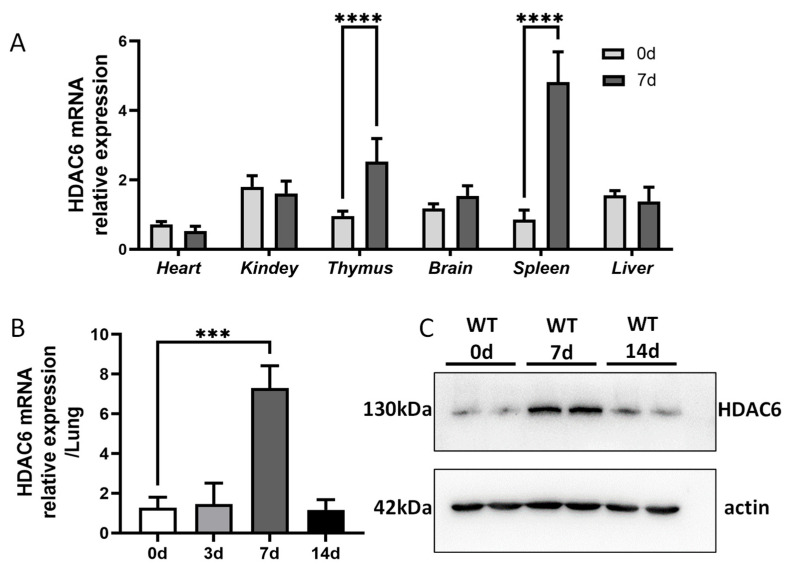
HDAC6 is upregulated in both immune organs and the infection site during *C. muridarum* infection. C57BL/6 mice were intranasally infected with 1 × 10^3^ inclusion-forming units (IFUs) of *C. muridarum*. Lung tissues and other organs were collected at the indicated time points post-infection (p.i.). (**A**,**B**) *HDAC6* mRNA expression in heart, kidney, thymus, brain, spleen, liver and lung tissues was measured by qPCR. (**C**) HDAC6 protein levels in lung tissues at 0, 7, and 14 days p.i. were detected by Western blot. Statistical significance of differences was determined by one-way ANOVA (*** *p* < 0.001; **** *p* < 0.0001).

HDAC6’s role in α-tubulin deacetylation is well-established, but its immunomodulatory mechanisms during bacterial infections are poorly characterized. We examined the mRNA expression of HDAC6 in various tissues of WT mice at baseline (uninfected) and on day 7 post-infection (p.i.) to evaluate their response to *C. muridarum* infection. Strikingly, *HDAC6* mRNA levels were significantly elevated in immune organs (thymus and spleen) after *C. muridarum* infection, with no statistically significant differences detected in non-immune tissues (heart, kidney, brain, and liver) (Figure 1A). We therefore analyzed HDAC6 expression in pulmonary tissues and observed a significant upregulation, with mRNA levels increasing from 3 days p.i., peaking at 7 days p.i., and declining by 14 days p.i. (Figure 1B). Consistently, HDAC6 protein expression was higher at 7 days p.i., and decreased by 14 days p.i. (Figure 1C).

These findings collectively demonstrate that HDAC6 participates in the host response to *C. muridarum* respiratory infection, showing significant induction in both immune organs and the infection site, with a particular upregulation in pulmonary tissues during the mid-phase of infection.

### 2.2. HDAC6 Enhances Host Defense Against C. muridarum Infection

**Figure 2:**
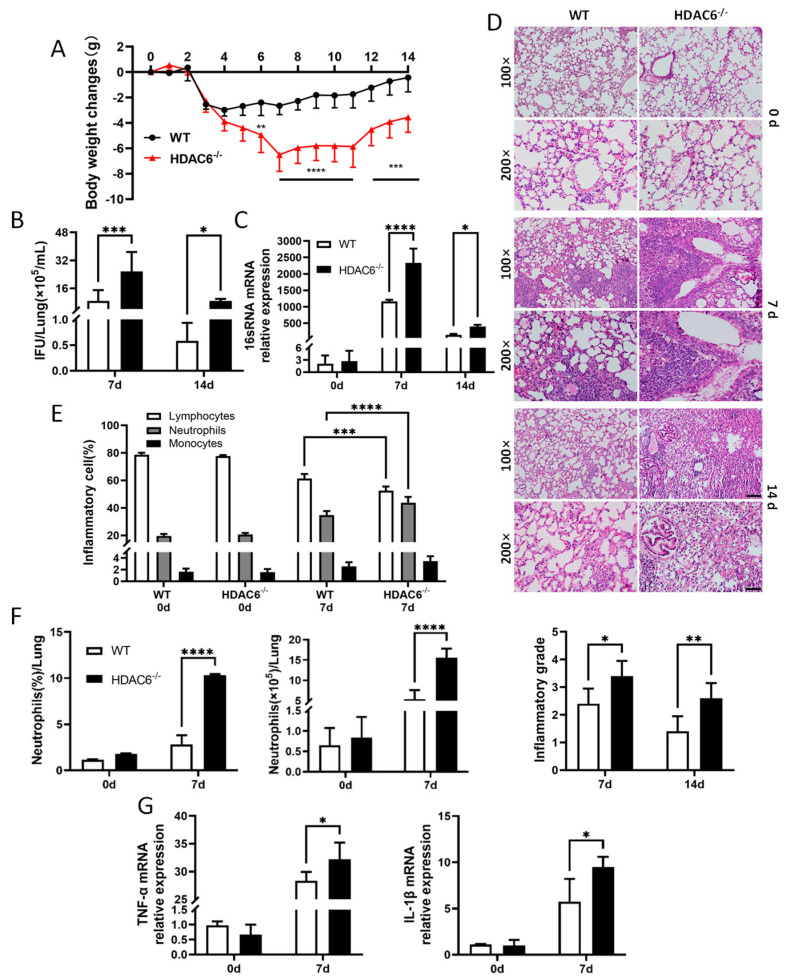
HDAC6 enhances host resistance following *C. muridarum* infection. C57BL/6 WT and HDAC6^−/−^ mice were intranasally infected with 1 × 10^3^ inclusion-forming units (IFUs) of *C. muridarum*. Lung tissues were collected at indicated time p.i. (**A**) Body weight changed in WT and HDAC6^−/−^ mice were monitored daily for 14 days p.i. (**B**,**C**) Lung bacterial loads were determined by quantifying IFUs (**B**) and *16S rRNA* mRNA expression (**C**). (**D**) Representative images of H&E-stained lung tissue sections at 100× (scale bar, 200 μm) and 200× (scale bar, 100 μm) magnification. Pathological scores were calculated based on the degree of inflammatory cell infiltration, alveolar structure destruction, and interstitial edema. (**E**) The percentage of inflammatory cells in lung tissues assessed by Giemsa staining. (**F**) Flow cytometry analysis of the percentage and absolute counts of neutrophils in the lungs. (**G**) The mRNA expression of *tnf*−*α* and *il*−*1β* were detected by qPCR. Data are presented as *mean* ± *SD* from *n* = 3–5 mice per genotype and time point, representative of one of three independent experiments (all data from the two additional independent replicate experiments are available in the Supplementary Material Table S1). Statistical significance of differences was determined by two-way ANOVA (* *p* < 0.05; ** *p* < 0.01; *** *p* < 0.001; **** *p* < 0.0001).

We used a *C. muridarum* respiratory infection model with WT and HDAC6-deficient (HDAC6^−/−^) mice to explore the role of HDAC6 in host defense against *Chlamydia* infection. The results showed that HDAC6^−/−^ mice exhibited more severe disease progression compared to WT mice during *C. muridarum* respiratory infection. Both groups of mice began losing weight from 3 to 7 days p.i., while HDAC6^−/−^ mice displayed a greater magnitude of weight reduction and protracted recovery, even failing to regain pre-infection weight by 14 days p.i. (Figure 2A). This prolonged weight loss in HDAC6^−/−^ mice directly reflected impaired bacterial clearance, with significantly higher IFU counts and elevated 16S rRNA mRNA expression in the lungs at 7 days p.i., and persistently higher bacterial loads through 14 days p.i. (Figure 2B,C). Histopathological analysis revealed more severe pulmonary damage in HDAC6^−/−^ mice, characterized by exacerbated inflammatory cell infiltration, alveolar wall destruction, and higher pathology scores (Figure 2D). Additionally, Giemsa staining confirmed increased granulocyte percentages in HDAC6^−/−^ mice at 7 days p.i. (Figure 2E). Neutrophils showed significantly elevated percentages and absolute numbers of neutrophils (CD45^+^CD11b^+^Ly6G^+^) in HDAC6^−/−^ mouse lungs at 7 days p.i. (Figure 2F). Consistently, the mRNA levels of pro-inflammatory cytokines *tnf-α* and *il-1β* were substantially upregulated in HDAC6^−/−^ mice (Figure 2G). These results demonstrate that HDAC6 plays a critical role in suppressing *C. muridarum* respiratory infections by enhancing pathogen clearance and host defense.

### 2.3. HDAC6 Suppresses Th2-Mediated Immunopathology During C. muridarum Lung Infection

**Figure 3:**
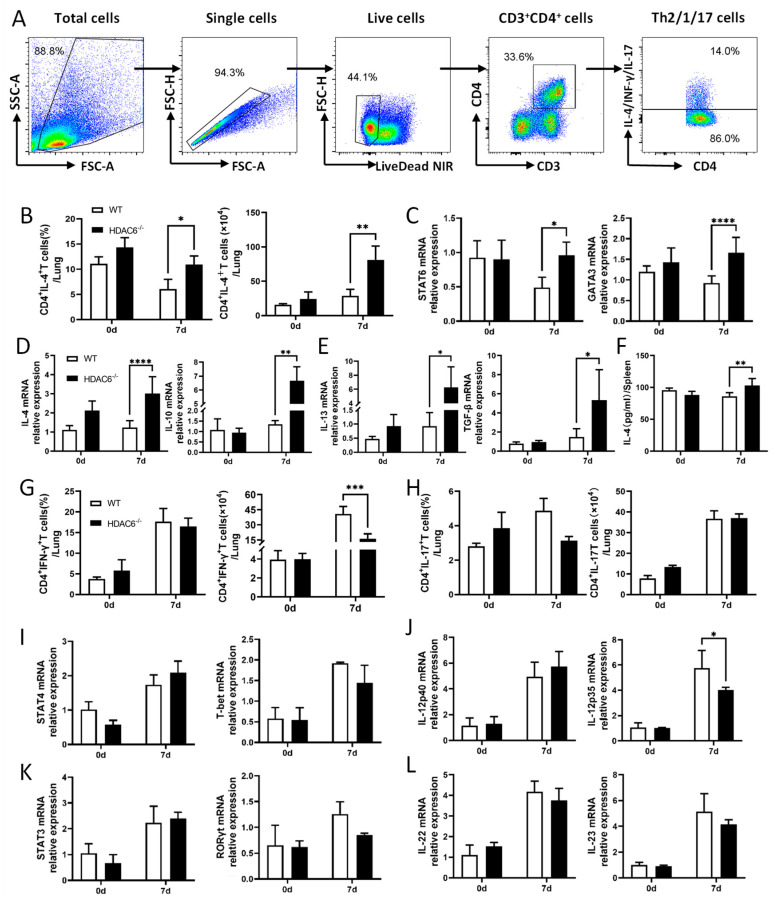
HDAC6 restrains IL-4 and Th2 transcription factors during *C. muridarum* infection. (**A**) Flow cytometry gating strategy for lung T helper cells. Live cells were selected based on FSC/SSC parameters, followed by the exclusion of doublets or dead cells. CD4^+^ T cells were identified as CD3^+^CD4^+^ cells, which were subsequently analyzed by intracellular cytokine staining to define Th2 cells (CD3^+^CD4^+^IL-4^+^ T cells), Th1 cells (CD3^+^CD4^+^IFN-γ^+^ T cells) and Th17 cells (CD3^+^CD4^+^IL-17^+^ T cells). (**B**,**G**,**H**) Flow cytometry data are summarized to show the percentages (above) and absolute numbers (below) of Th2(B), Th1(G), and Th17(H) cells in the lungs of WT and HDAC6^−/−^ mice on days 0 and 7 p.i. (**C**,**I**,**K**) Relative mRNA expression of Th2 transcription factors (*stat6*, *gata3*), Th1 transcription factors (*stat4*, *T-bet*), and Th17 transcription factors (*stat3*, *RORγt*) in lung tissues was measured by qPCR. (**D**,**E**,**J**,**L**) Relative mRNA expression of Th2-associated cytokines (*il-4*, *il-10*, *il-13*, *TGF-β*), Th1-associated cytokines (*il-12p40*, *il-12p35*,) and Th17-associated cytokines (*il-22*, *il-23*) in lung tissues was measured by qPCR. (**F**) IL-4 levels in spleen cell culture supernatants were quantified by ELISA. Data are presented as *mean* ± *SD* from *n* = 3–5 mice per genotype and time point, representative of one of three independent experiments (raw data from the two additional independent replicate experiments are available in the Supplementary Material Table S2). Statistical significance of differences was determined by two-way ANOVA (* *p* < 0.05; ** *p* < 0.01; *** *p* < 0.001; **** *p* < 0.0001).

T cell-mediated immunity, particularly IFN-γ-secreting Th1-type responses, plays a pivotal protective role in host defense against respiratory *C. muridarum* infection [25], whereas excessive Th2-mediated responses are known to compromise host resistance [26,27]. Our results showed that, compared to WT mice, HDAC6^−/−^ mice displayed increased percentages and absolute numbers of Th2 cells (CD4^+^ IL-4^+^) following respiratory *C. muridarum* infection (Figure 3A,B). Given that STAT6-GATA3 signaling is essential for Th2 cell differentiation, further analysis using qPCR showed that infected WT mice exhibited decreased mRNA expression of Th2-related transcription factors (*stat6*, *gata3*) and cytokines (*il-4*, *il-10*, *TGF-β*, *il-13*) on day 7 p.i., and HDAC6^−/−^ mice displayed elevated expression of these factors compared to infected WT mice (Figure 3C–E). Consistently, ELISA confirmed higher IL-4 secretion by splenocytes from HDAC6^−/−^ mice (Figure 3F). In contrast, no significant differences were observed in either the percentages or absolute numbers of Th1 (CD4^+^ IFN-γ^+^) or Th17 (CD4^+^ IL-17^+^) cells in lung tissues between HDAC6^−/−^ and WT mice (Figure 3G,H). Accordingly, mRNA expression levels of their key transcriptional regulators (*stat4*, *T-bet*, *stat3*, *RORγt*) and associated cytokines (*il-12p40*, *il-12p35*, *il-22*, *il-23*) showed no difference between infected groups (Figure 3I–L).

These findings reveal that HDAC6 selectively suppresses Th2 cell-mediated immune responses in the lungs during *C. muridarum* infection without significantly impacting Th1 or Th17 cell responses. Th2-mediated imbalance disrupts adaptive immunity, aggravating pulmonary inflammation and tissue damage.

### 2.4. HDAC6 Restrains Macrophage Infiltration and M2 Polarization During C. muridarum Infection

**Figure 4:**
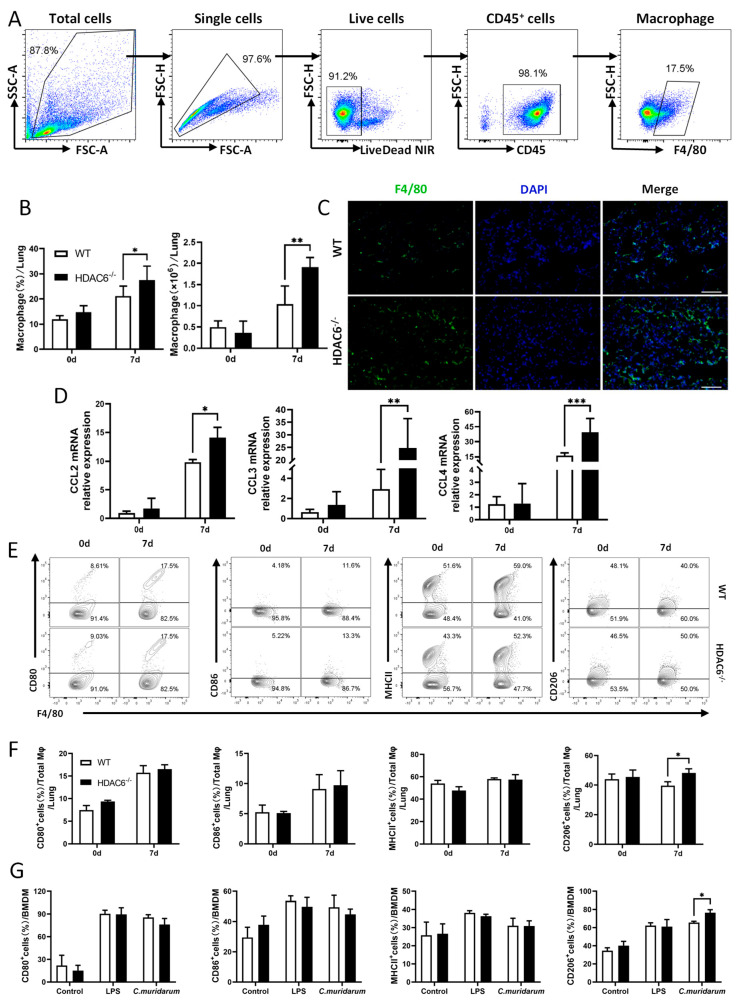
HDAC6^−/−^ mice promote macrophage infiltration and M2 macrophage polarization following *C. muridarum* infection. (**A**) Flow cytometry gating strategy for lung immune cells. Live cells were selected based on FSC/SSC parameters, followed by exclusion of doublets or dead cells. Macrophages (Mφ) were identified as CD45^+^F4/80^+^ cells. (**B**) Representative flow cytometry bar graphs showing the percentage and absolute counts of Mφ in the lungs of WT and HDAC6^−/−^ mice on day 7 p.i. (**C**) Representative immunofluorescence images of frozen lung sections from WT and HDAC6^−/−^ mice on day 7 p.i. (green: F4/80^+^ cells; blue: DAPI) (200×), scale bar: 100 µm. (**D**) The mRNA expression of macrophage chemotactic factors *ccl2* (left), *ccl3* (middle), and *ccl4* (right) in lung tissues was measured by quantitative real-time PCR (qPCR). (**E**–**G**) Representative flow cytometry plots (**E**) and bar graphs showing the percentage and absolute counts of CD80^+^, CD86^+^, MHC II^+^, and CD206^+^ cells within CD45^+^F4/80^+^ Mφ (**F**) and BMDMs (**G**) from lung tissues on day 7 p.i. Data are presented as *mean* ± *SD* from *n* = 3–5 mice per genotype and time point, representative of one of three independent experiments (raw data from the two additional independent replicate experiments are available in the Supplementary Material Table S3). Statistical significance of differences was determined by two-way ANOVA (* *p* < 0.05; ** *p* < 0.01; *** *p* < 0.001).

Next, we focused on macrophages, a pivotal regulator of T cell polarization [28], which can differentiate into M1 or M2 phenotypes depending on the microenvironment. M1 macrophages recruit Th1 cells via secreted chemokines to create a bactericidal environment for pathogen clearance, while M2 macrophages recruit Th2 cells via secreted chemokines to modulate the immune response [29,30]. We hypothesized that HDAC6 deficiency drives Th2-mediated immune responses, potentially through altered macrophage-mediated regulation of T cell differentiation. The results showed that at day 7 p.i., pulmonary macrophages (CD45^+^F4/80^+^) in HDAC6^−/−^ mice exhibited significantly enhanced infiltration compared to WT mice (Figure 4A,B). Immunofluorescence staining further confirmed enhanced F4/80 expression in HDAC6^−/−^ mice lung tissues (Figure 4C) and elevated mRNA expression of macrophage chemokines *ccl2*, *ccl3*, and *ccl4* was also observed in HDAC6^−/−^ mice. (Figure 4D). Our results showed no significant differences in the expression of M1 markers (CD80^+^, CD86^+^, MHC II^+^) between infected HDAC6^−/−^ and WT mice. However, HDAC6^−/−^ mice exhibited an increased trend in the expression of M2 markers (CD206^+^) compared to WT mice at 7 days p.i. (Figure 4E,F). The vitro experiments also showed that HDAC6^−/−^ bone marrow-derived macrophages (BMDMs) exhibited an enhanced M2 polarization after *C. muridarum* stimulation, while there were no significant differences in M1 polarization (Figure 4G).

Collectively, these findings demonstrate that HDAC6 restrains excessive macrophage infiltration in lung tissues and suppresses M2 polarization during *C. muridarum* infection.

### 2.5. HDAC6 Inhibits the Activation of M2 During C. muridarum Infection

**Figure 5:**
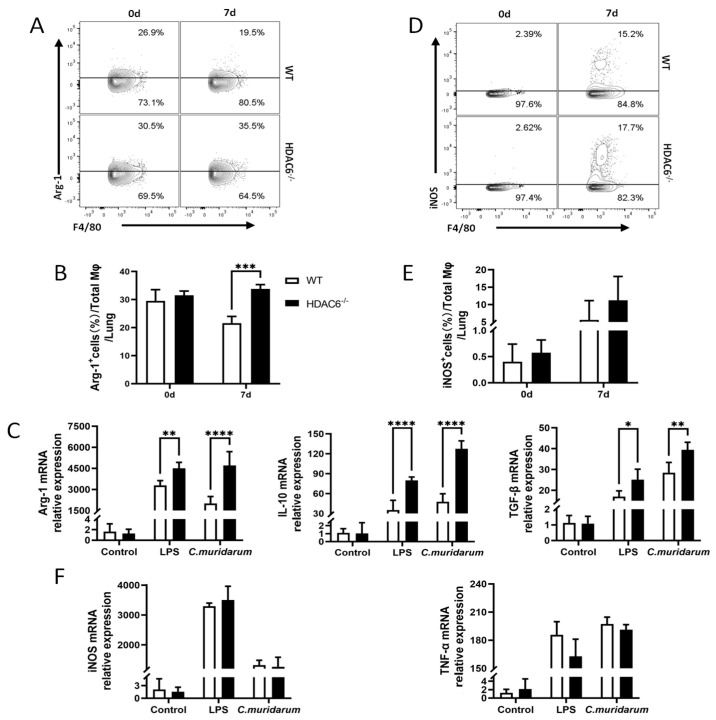
HDAC6 deficiency enhances M2 macrophage function following *C. muridarum* infection. (**A**,**D**) Representative flow cytometry plots of Arg−1^+^ (**A**) and iNOS^+^ (**D**) cells within total Mφ from lung tissues of WT and HDAC6^−/−^ mice at day 7 p.i. (**B**,**E**) Bar graphs summarize the percentages of Arg−1^+^ (**B**) and iNOS^+^ (**E**) cells within total Mφ. (**C**,**F**) BMDMs from WT and HDAC6^−/−^ mice were stimulated with *C. muridarum* or LPS for 24 h. The mRNA expression of *Arg*−*1*, *il*−*10*, *TGF*−*β*, *iNOS* and *tnf*−*α* was measured by qPCR. Data are presented as *mean* ± *SD* from *n* = 3–5 mice per genotype and time point, representative of one of three independent experiments (raw data from the two additional independent replicate experiments are available in the Supplementary Material Table S3). Statistical significance of differences was determined by two-way ANOVA (* *p* < 0.05; ** *p* < 0.01; *** *p* < 0.001; **** *p* < 0.0001).

The polarization of M1 and M2 macrophages is closely intertwined with their activation, a relationship pivotal to regulating inflammatory and immune processes. To assess macrophage activation following *C. muridarum* infection, we analyzed the expression of Arg-1, a key marker of M2 macrophage activation, given that the balance between M1 and M2 polarization states is crucial for immune regulation. We observed that Arg-1 expression in lung F4/80^+^ macrophages was significantly elevated in HDAC6^−/−^ mice at day 7 p.i. (Figure 5A,B). Further validation using BMDMs showed that, following *C. muridarum* infection, the *Arg-1* mRNA level was significantly higher in HDAC6^−/−^ BMDMs than in WT BMDMs, along with elevated expression of M2-associated cytokines (*TGF-β, il-10*) in HDAC6^−/−^ BMDMs. (Figure 5C). In contrast, iNOS—a hallmark of M1 macrophage activation that plays a critical role in various inflammatory responses [31]—showed no significant difference in expression between WT and HDAC6^−/−^ mice, either in lung F4/80^+^ macrophages or BMDMs, nor did M1-associated TNF-α levels (Figure 5D–F).

These findings demonstrate that HDAC6 restrains M2 polarization by downregulating M2-associated effectors (Arg-1, IL-10, and TGF-β) that would potentially drive Th2 cell differentiation. This balanced regulation of the M1 and M2 polarization supports host defense during *C. muridarum* infection.

### 2.6. HDAC6 Inhibits Th2 Differentiation via Macrophage-Mediated T Cell Regulation During C. muridarum Infection

**Figure 6:**
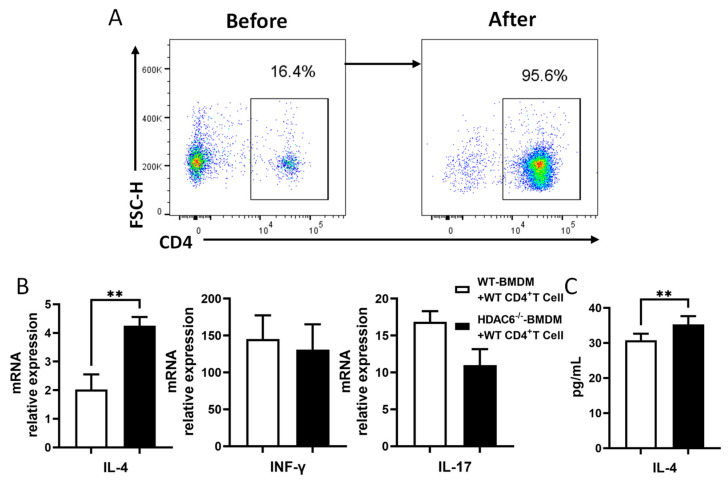
HDAC6 deficiency promotes Th2 differentiation through macrophage–T cell interactions during *C. muridarum* infection. (**A**) Purity analysis of CD3^+^CD4^+^ T cells isolated from spleen cells of naïve WT mice via magnetic-activated cell sorting (MACS) (95.6% pure). (**B**) The mRNA relative expressions of *il-4*, *ifn-γ* and *il-17* mRNA in CD4^+^ T cells were quantified by qPCR after co-culture with *C. muridarum*-stimulated BMDMs. (**C**) ELISA analysis of IL-4 secretion in supernatants from CD4^+^ T cell co-culture with *C. muridarum*-stimulated BMDMs. Data are presented as *mean* ± *SD* from *n* = 3–5 mice per genotype and time point, representative of one of three independent experiments. Statistical significance of differences was determined by one-way ANOVA (** *p* < 0.01).

T cell–macrophage interactions are critical for immunoregulatory crosstalk in antimicrobial immunity and inflammation. Our previous findings have demonstrated that HDAC6 deficiency enhances Th2 responses and M2 macrophage activation during *C. muridarum* infection. To investigate whether HDAC6-mediated Th2-associated immunopathology depends on macrophages, we performed co-culture experiments using CD4^+^ T cells (95.6% purity) isolated from the spleens of naïve WT mice (Figure 6A), together with BMDMs from WT or HDAC6^−/−^ mice that had been stimulated with *C. muridarum* for 24 h. We observed that HDAC6^−/−^ BMDMs significantly increased IL-4 mRNA levels in CD4^+^ T cells compared to WT BMDMs, whereas IFN-γ and IL-17 levels remained unchanged (Figure 6B). Consistent results were obtained in ELISA analysis of co-culture supernatants, which showed that WT CD4^+^ T cells co-cultured with HDAC6^−/−^ BMDMs produced significantly higher levels of IL-4 (Figure 6C). These findings indicate that HDAC6 restrains Th2 differentiation by regulating macrophage polarization, which in turn contributes to protective immunity during *C. muridarum* infection.

## 3. Discussion

HDAC6 exhibits dual regulatory roles in infectious diseases, with context-dependent functions as evidenced by previous studies. In *M. tuberculosis* infection, HDAC6 enhances bacterial clearance by augmenting macrophage phagocytic activity [32], whereas during *L. monocytogenes* infection, it restricts excessive inflammation through the suppression of STAT4-K667 phosphorylation [16]. In LPS-induced acute lung injury, HDAC6 inhibition attenuates inflammatory responses by blocking IĸB phosphorylation and NF-ĸB activation, thereby reducing inflammasome activity [15]. Notably, *Legionella pneumophila* infection significantly upregulates HDAC6 expression in murine lung tissues [33]. In our current study, we similarly observed the significant induction of HDAC6 expression during *C. muridarum* infection, where it appears to play a protective immunomodulatory role. HDAC6 deficiency resulted in exacerbated disease manifestations, including more pronounced weight loss, delayed recovery, impaired bacterial clearance, aggravated pulmonary pathology with enhanced inflammatory cell infiltration, and the elevated expression of the proinflammatory cytokines TNF-α and IL-1β. In the context of respiratory *C. muridarum* infection, effective host defense entails not only the eradication of the pathogen but also the restraint of disproportionate immunopathology to preserve tissue integrity. Our data demonstrate that HDAC6 deficiency simultaneously impairs both aspects: it delays bacterial clearance and exacerbates lung injury. This indicates that the primary protective mechanism of HDAC6 is the maintenance of immune homeostasis by constraining the excessive Th2-M2 axis. These findings not only expand our understanding of HDAC6’s regulatory mechanisms in infectious diseases but also provide novel insights for developing therapeutic strategies against chlamydial infection, highlighting the crucial protective role of HDAC6 in host defense against *C. muridarum*.

Th2 cells play a pivotal role in immunopathological processes by orchestrating immune responses through their signature cytokine IL-4. Elevated Th2 responses during *M. tuberculosis* infection have been shown to suppress Th1-mediated protective immunity, thereby promoting bacterial persistence in the host [34]. In the context of Chlamydia infection, our understanding of the immunological cascade reveals that IL-17 production is initially dominated by γδT cells during early infection, with Th17 cells subsequently assuming this role in later stages to mediate protective immunity [35,36]. Yoojung Kwon [37] provided compelling evidence of HDAC6 transcriptional upregulation and an increased secretion of characteristic Th2 cytokines IL-4, IL-5, IL-6, and IL-13 in an atopic dermatitis mouse model. While the involvement of HDAC6 in Th cell differentiation during bacterial infections remains poorly characterized, our current study provides compelling evidence that HDAC6 deficiency leads to enhanced Th2 polarization in infected lungs, as manifested by the upregulated expression of key transcriptional factors (STAT6 and GATA3) and effector cytokines (IL-4, IL-10, TGF-β, and IL-13), without significantly altering Th1 and Th17 differentiation. We propose that HDAC6 may selectively regulate Th2 differentiation through acetylation modulation of specific transcription factors like GATA3 and STAT6, while exerting minimal effects on Th1 and Th17 pathways. The dominant Th1 and Th17 differentiation during Chlamydia infection may potentially obscure HDAC6-mediated regulatory effects, or Th1 and Th17 differentiation may rely on compensatory mechanisms involving other HDAC family members, thereby maintaining lineage stability despite HDAC6 deficiency. Importantly, our findings establish that HDAC6 serves as a critical negative regulator of Th2-mediated pulmonary immunopathology during respiratory chlamydial infection by suppressing GATA3 or STAT6 activation and subsequent Th2 cytokine production, highlighting its unique role in Th2-specific immunomodulation. Notably, the unimpaired Th1 and Th17 responses in HDAC6^−/−^ mice suggest that pathogen clearance failure is not due to a lack of protective immunity, but likely results from its functional suppression by a dominant immunosuppressive milieu. The robust Th2-M2 response, characterized by elevated IL-4, IL-10, and TGF-β, may create a microenvironment that antagonizes Th1 effector functions and directly compromises macrophage bactericidal activity, ultimately tipping the balance towards pathogen persistence and tissue damage. However, the precise molecular mechanisms underlying HDAC6-mediated regulation warrant further investigation.

Macrophages, as pivotal effector cells of the innate immune system, play central roles in host defense, antigen presentation, and immunomodulation, exhibiting remarkable plasticity to differentiate into functionally distinct subsets in response to the immune microenvironment [38]. Classically activated M1 macrophages, primarily induced by IFN-γ, are characterized by a high expression of iNOS, IL-12, and TNF-α, demonstrating potent pathogen-killing capacity and pro-inflammatory properties. In contrast, alternatively activated M2 macrophages, driven by Th2 cytokines such as IL-4 and IL-13, express elevated levels of Arg-1 and contribute to anti-inflammatory responses, tissue repair, and immunoregulation [39]. Emerging evidence positions HDAC6 as a critical regulator of macrophage polarization, as demonstrated by its role in promoting M2 polarization through the IL-10/STAT6 axis in endometriosis-associated ovarian carcinoma [40], while HDAC6 inhibitors have been shown to suppress iNOS expression in septic shock models [41] and enhance M2-associated IL-10 production in a chemotherapy-induced peripheral neuropathy model [42].

T cell-derived cytokines direct macrophage polarization, and polarized macrophages in turn modulate T cell function through antigen presentation and cytokine secretion. Studies have demonstrated that during tissue repair processes, Tregs can directly induce macrophage polarization toward the M2 phenotype through IL-10 secretion, thereby modulating the liver regeneration microenvironment [43]. Correspondingly, T cell functional deficiencies similarly impact macrophage polarization, as evidenced by BAP31 deficiency impairing CD4^+^ T cell activation and consequently reducing IFN-γ and IL-4 production, which affects macrophage polarization [44]. In tumor microenvironments, HPV-induced M2-polarized TAMs facilitate cervical cancer immune evasion through Th1 response suppression [45]. Our current study demonstrates that HDAC6 deficiency in the *C. muridarum* infection model results in significantly enhanced pulmonary macrophage infiltration with pronounced M2 polarization, as evidenced by the upregulated CD206 expression and increased secretion of M2 markers (Arg-1 and IL-10), which were further validated in vitro using HDAC6^−/−^ -BMDMs. This M2-dominant phenotype correlates strongly with exacerbated Th2 responses observed in vivo, suggesting that HDAC6 normally serves as a negative regulator of M2 polarization through STAT6 deacetylation. The absence of HDAC6-mediated regulation leads to uncontrolled M2 activation, creating a pathogenic positive feedback loop through the sustained production of Th2-driving cytokines (IL-4, IL-10), which not only impairs pathogen clearance but also disrupts macrophage phenotypic equilibrium. Notably, our in vitro experiments demonstrated that CD206 expression was significantly upregulated in HDAC6^−/−^ BMDMs following *C. muridarum* stimulation. While this finding appears inconsistent with early studies suggesting Chlamydia fails to polarize unpolarized macrophages into canonical M1/M2 phenotypes, it underscores the critical role of host epigenetic context in pathogen–macrophage crosstalk. Recent studies have revised this traditional view, establishing that Chlamydia and other intracellular bacteria regulate macrophage polarization not via an “all-or-nothing” mechanism but in a host signaling network-dependent manner. For example, Radziej [46] utilized dual isotopologue profiling to show *C. trachomatis* replicates efficiently in human M2-like macrophages yet is restricted in M1-like subsets; Moldovan [47] further identified that *C. trachomatis* exploits sphingolipid metabolism in M2-like macrophages for intracellular proliferation. This paradigm extends to other pathogens: *Salmonella* induces M2 polarization via SteE-mediated STAT3 activation [48], and even the traditionally extracellular *Staphylococcus aureus* drives M2 polarization to establish chronic intracellular infection in osteomyelitis [49]. Herein, HDAC6 deficiency—abrogating the host’s intrinsic restraint on M2 polarization—enables macrophages to exhibit pronounced M2 polarization upon Chlamydia stimulation. Of note, *Chlamydia* are able to survive and replicate freely in M2 macrophages, which may further impair pathogen clearance and exacerbate infection-induced immunopathology [50,51], ultimately exacerbating Th2-skewed immunopathology. These findings establish HDAC6 as a selective modulator of M2 polarization with profound implications for Th2 immune responses, while highlighting the complexity of macrophage–T cell interactions in infectious disease pathogenesis.

Macrophages, as professional antigen-presenting cells, play a pivotal role in immune responses through direct cellular interactions with CD4^+^ T cells. Our co-culture experiments revealed that direct cell-to-cell contact between HDAC6^−/−^ BMDMs and CD4^+^ T cells is necessary to promote Th2 differentiation during *C. muridarum* infection, indicating that physical intercellular interactions are the critical driver in the Th2 differentiation process. Intriguingly, this enhanced Th2 response occurred even though MHC II expression was comparable between HDAC6^−/−^ and WT macrophages—suggesting the involvement of non-classical antigen-presenting pathways in this regulatory process. This phenomenon has precedents in other infection or disease contexts: in *M. tuberculosis* infection, macrophage–T cell contact enhances antibacterial function via SLAMF1 signaling [52]; in tumor microenvironments, a subset of CD4^+^ T cells exerts antitumor effects independently of MHC expression [53]. We thus propose that HDAC6 likely modulates Th2 differentiation through the regulation of STAT6 activation or components of the macrophage–T cell immune synapse, though the exact MHC II-independent mechanisms require further validation. These findings shed new light on the underappreciated role of direct cell contact in immune regulation during chlamydial infection, emphasizing that beyond classical MHC II-TCR signaling, the molecular composition and signaling intensity at the macrophage–T cell interface are decisive factors shaping T cell differentiation’s fate.

Our study is the first to establish a direct link between HDAC6 and chlamydial respiratory infection, uncovering its indispensable immune protective role in the *C. muridarum* lung infection model. We further report the novel finding that HDAC6^−/−^ macrophages undergo marked M2 polarization in the chlamydial infection microenvironment, which may act as a key driver of post-infection M2-Th2 axis hyperactivation. Additionally, our in vitro macrophage–T cell co-culture assays reveal for the first time that HDAC6^−/−^ macrophages directly induce Th2 cell differentiation, identifying a novel mode of macrophage–T cell crosstalk that modulates immune responses during chlamydial infection—thus defining HDAC6 as a critical negative regulator of the M2-Th2 axis in this infection context. While our study establishes HDAC6 as a critical negative regulator of the Th2-M2 axis during chlamydial respiratory infection, providing novel insights into its protective immunomodulatory role in maintaining immune homeostasis, several key limitations and unresolved questions merit acknowledgment and future investigation. First, the direct deacetylase substrate of HDAC6 mediating the regulation of the Th2-M2 axis remains elusive. Based on our observations of heightened STAT6/GATA3 signaling in HDAC6^−/−^ mice and consistent with the prior literature linking HDAC6 to STAT6-mediated immune cell polarization, STAT6 and/or GATA3 emerge as the most plausible direct targets. However, definitive evidence for the direct physical interaction between HDAC6 and these transcription factors, as well as rigorous verification of the HDAC6-mediated deacetylation of specific lysine residues within STAT6/GATA3, is still lacking. Second, the specific molecular mechanisms by which HDAC6 modulates the STAT6/GATA3 pathway, including how deacetylation alters the transcriptional activity, nuclear translocation, or protein stability of these factors, remain incompletely elucidated. Future studies integrating cell type-specific knockout models, site-directed acetylation mutants, and co-immunoprecipitation coupled with mass spectrometry will be pivotal to deciphering this precise molecular cascade. These investigations will refine our understanding of HDAC6-mediated immune regulation and lay a more solid foundation for developing targeted immunotherapies against chlamydial infections and other immune-imbalanced inflammatory diseases.

## 4. Materials and Methods

### 4.1. Mice

Female mice weighing 18–22 g were used in the experiments. Wild-type (WT) C57BL/6 mice were purchased from Beijing Huafukang Biotechnology (Production License No. SCXK (Jing) 2019-005). HDAC6 knockout (HDAC6^−/−^) mice developed on a C57BL/6 genetic background were generously supplied by Dr. Jun Zhou (Nankai University). The animals were housed and cared for at the Laboratory Animal Center of Tianjin Medical University under specific pathogen-free (SPF) conditions. Housing management as well as the experimental process were performed in compliance with the Institutional Animal Care and Use Committee of Tianjin Medical University’s ethical recommendations and approved by the committee (Approval No. SYXK (Jin) 2019-0004).

### 4.2. C. muridarum Respiratory Infection Mouse Model

The *C. muridarum* respiratory infection model was developed as follows: mice were anesthetized using isoflurane inhalation and then inoculated intranasally with 40 μL of *C. muridarum* suspension at a concentration of 1 × 10^3^ inclusion-forming units (IFU). All the experiments were conducted in a Biosafety Level 1 (BSL-1) laboratory [54].

### 4.3. Quantification of Chlamydia Inclusion-Forming Units (IFUs)

Homogenization of the lung tissue from *C. muridarum*-infected mice was done with a tissue grinder (DWK Life Sciences, Millville, NJ, USA) on ice. Centrifuging the homogenate was followed by harvesting the supernatant and the subsequent infection of the HeLa-299 cells, which were incubated at 37 °C in humidified 5% CO_2_ incubator for 2 h. The cells without supernatant were then cultured in high-glucose Dulbecco modified Eagle medium (DMEM, Gibco, Grand Island, NY, USA) with 10% fetal bovine serum (FBS) for 36–40 h. The cell cultures were thereafter treated with ice-cold methanol, and sequentially incubated with mouse anti-Chlamydia LPS IgG (Invitrogen, Carlsbad, CA, USA) (70 min) and (HRP)-conjugated goat anti-mouse IgG (Solarbio, Beijing, China) (50 min). 4-chloro-1-naphthol (4-CN, Solarbio Life Science, Beijing, China) was used to perform chromogenic reactions, and inclusion bodies were quantified through observation using a light microscope to assess their number as IFUs [54].

### 4.4. Lung Tissue Histopathological Staining

The lung tissues were removed and dissected out of *C. muridarum*-infected mice at 0, 7, and 14 days p.i. The lung tissues were put in 4% paraformaldehyde (PFA) and allowed to fix overnight (48 h), followed by a series of processing procedures such as dehydration of the tissues in gradient ethanol, clearing in xylene, embedding in paraffin and sectioning them into 4 μm thick histological sections. After staining with hematoxylin and eosin (H&E), the sections were observed using a light microscope to assess the pathological changes in the lung tissues.

### 4.5. Immunofluorescence Staining

On day 7 p.i., lung tissues were fixed in 4% paraformaldehyde (PFA) at 4 °C, then dehydrated in 15% and 30% sucrose solutions in sequence during the next twenty-four hours. Samples were put in Tissue-Tek O.C.T. Compound (SAKURA, Baltimore, MD, USA), and the cryosections obtained were 8 μm thick. The sections were incubated with the 10% goat serum at room temperature for 1 h and subsequently exposed to F4/80 antibody (1:200 dilution, abcam, Cambridge, UK) overnight at 4 °C. Thereafter, the sections were incubated at room temperature with fluorescent-labeled secondary antibody Goat Anti-Rat IgG H&L (Alexa Fluor 488, 1:300 dilution, abcam, Cambridge, UK) in the dark. After they had been washed, they were stained with Auto-FluoQuencher (APPLYGEN, Beijing, China) for 15 min and were stained with DAPI Fluoromount-G (SouthernBiotech, Birmingham, AL, USA). Images were acquired using a fluorescent microscope at 200× magnification [54].

### 4.6. Wright–Giemsa Staining

The single-cell suspensions of the lung tissues were prepared on day 7 p.i. for the uninfected and *C. muridarum* infected WT and HDAC6^−/−^ mice. An individual 10 μL sample of the suspension was spread on the glass microscope slide and dried at room temperature for 30 min. To stain the smears, we used the Wright–Giemsa Stain Kit (Baso Biotechnology, Zhuhai, China). All experimental procedures were performed by strictly following the operating instructions of the kit. The slides were analyzed using a light microscope to count cells.

### 4.7. Preparation of Single-Cell Suspensions from Spleen and Lung Tissues

Spleen and lung tissues were collected under sterile conditions from mice at 0 and 7 days after *C. muridarum* respiratory infection. Spleens were gently homogenized and filtered through a 200-mesh nylon sieve. Collagenase XI (Sigma-Aldrich, Darmstadt, Germany, 1 mg/mL) was used to digest lung tissues at 37 °C for 55 min and EDTA (1 mM) was then added to inhibit cell clumping. A mixture of 35% Percoll (Sigma-Aldrich, Darmstadt, Germany) and red blood cell lysis buffer was used to eliminate tissue debris and erythrocytes. The cells were recovered in RPMI 1640 media (Gibco, Grand Island, NY, USA) containing 2% FBS. Trypan blue exclusion assay was used to measure cell viability where viable cells were denoted as trypan blue-negative. The densities of lung cells were adjusted to 2 × 10^6^ cells/mL and splenic cell densities to 1 × 10^7^ cells/mL before further experimental assays [54].

### 4.8. Bone Marrow-Derived Macrophage (BMDM) Induction

Skeletal bone marrow cells were aseptically excised out of the tibias and femurs of naive mice. After flushing the bone marrow cavity with ice-cold PBS to retrieve the cells, red blood cells were lysed with ACK lysis buffer. The cells were resuspended in complete medium supplemented with 20 ng/mL M-CSF [55] (SinoBiological, Beijing, China) and seeded into a 6-well plate at a density of 2 × 10^6^ cells/well. Fresh medium with M-CSF was replaced on days 3, 5 and 7. Mature BMDMs were harvested on day 7 with *C. muridarum* stimulation (MOI = 10) or lipopolysaccharide (LPS; 100 ng/mL) (Sigma, Burlington, MA, USA) stimulation after 24 h. The cells were subsequently subjected to further experiments [56].

### 4.9. Flow Cytometry Analysis

Surface staining: Lung single-cell suspensions or BMDMs were incubated with the Zombie NIR Fixable Viability Kit (423106, BioLegend, San Diego, CA, USA) at room temperature in the dark as a viability marker to avoid including dead cells. The cells were next washed with 2% FBS-PBS and then incubated with CD16/CD32 (Invitrogen, Carlsbad, CA, USA) in darkness at 4 °C to prevent non-specific Fc gamma receptor binding. Next, the following fluorochrome-conjugated antibodies (all purchased from BioLegend) were stained on the cells for 30 min at 4 °C in the dark: anti-CD45-PerCp-Cy5.5, anti-F4/80-APC, anti-CD11b-FITC, anti-Ly6G-APC, anti-CD11c-FITC, anti-CD80-PE, anti-CD86-PE-Cy7, anti-MHCII-PE, anti-CD206-PE, anti-iNOS-PE, and anti-Arg-1-PE-Cy7. Data from flow cytometry were obtained through the FACS Canto II flow cytometer (BD Biosciences, Franklin Lakes, NJ, USA) [54].

Intracellular staining: Mononuclear cells (1 × 10^6^ cells per well) of lung were plated in 48-well plates and activated with phosphol 12-myristate 13-acetate (PMA, 50 ng/mL, Solarbio, Beijing, China), ionomycin (1 μg/mL, Sigma-Aldrich, Darmstadt, Germany), and brefeldin A (BFA, 5 μg/mL, BioLegend, San Diego, CA, USA) at 37 °C in a 5% CO_2_ incubator over 5 h. Cells were subsequently stained with the viable stain Zombie NIR before being stained with the surface antibody anti-CD4-APC. To detect intracellular cytokines, cells were fixed, permeabilized, and stained with anti-IL-4-PE, anti-IFN-gamma-PE-Cy7, and anti-IL-17-PE. Data were collected using a FACS Canto II flow cytometer [54].

### 4.10. Western Blot Analysis

Protein samples were subjected to 10% SDS-PAGE and blotted to a PVDF membrane through a normal wet transfer system (filter paper to gel to PVDF membrane to filter paper). Upon completion of transfer, the membrane was incubated with 5% bovine serum albumin (BSA) blocking buffer at room temperature for 2 h. The membrane was subsequently incubated at 4 °C overnight with a primary antibody, rabbit anti-mouse HDAC6 (abcam), at a concentration of 1:2000 in TBST. Three washes with TBST at 5 min each were applied to the membrane and then the membrane was incubated with goat anti-rabbit IgG (Absin, Shanghai, China) conjugated with horseradish peroxidase (HRP), diluted to a factor of 1:5000 in TBST at room temperature for 2 h. A further three washes with TBST (5 min each) were performed and then the membrane was exposed to ECL Plus chemiluminescent substrate (Solarbio, Beijing, China) and viewed with Tanon5200 Chemiluminescent Imaging System (Tanon) [54].

### 4.11. Real-Time Quantitative PCR (qPCR)

Total RNA was extracted with Trizol reagent (Solarbio, Beijing, China) to extract total RNA from lung tissues or cells. RNA was reverse-transcribed to cDNA with the Script RT-PCR Kit (TransGen Biotech, Beijing, China). A LightCycler 96 machine (Roche, Basel, Switzerland) and SYBR Green qPCR Mix (GenStar, Beijing, China) were used to perform quantitative PCR. Each step involved was conducted exactly as per the recommendations of manufacturers. Relative gene expression levels were determined through the 2^−ΔΔCt^ approach. The primers used in this study were purchased from Shanghai Sangon Biotech, and the sequences are given in Table 1 [54].

### 4.12. UV Inactivation of C. muridarum

*C. muridarum* with a known concentration were transferred to a 6 mm cell culture dish and irradiated on ice in a clean bench. The UV irradiation was performed using a CL-1000 Ultraviolet Crosslinker with 254 nm UV-C bulbs, at a vertical distance of 10 cm between the dish and the UV source for 30 min, with an estimated fluence of approximately 120 J/cm^2^ (1200 J/m^2^). For the validation of complete inactivation, the treated *C. muridarum* suspension was inoculated into HeLa-299 cell and cultured for 48 h at 37 °C in a 5% CO_2_ incubator. The formation of chlamydial inclusions was detected by immunofluorescence staining, and the 100% inactivation of *C. muridarum* was confirmed when no visible chlamydial inclusions were observed under the microscope.

### 4.13. Co-Culture of BMDMs and CD4^+^ T Cells

To determine the effects of *C. muridarum*-stimulated macrophages on T cell responses, BMDMs were cultured in 200 µL complete medium in U-bottom 96-well plates at a density of 1 × 10^5^ cells/well. Next, BMDMs were activated by UV-inactivated *C. muridarum* (MOI = 10), which had been incubated at 37 °C in a humidified atmosphere of 5% CO_2_ to obtain an effect after 24 h of incubation. Spleen CD4^+^ T cells were purified from naive WT mice in accordance with the instructions of the MACS CD4^+^ T Cell Isolation Kit (Miltenyi Biotec, Bergisch Gladbach, Germany). The purified CD4^+^T cells were next cultured at a 5:1 ratio (T cells:BMDMs) with *C. muridarum*-stimulated BMDMs under the same incubator conditions in 200 μL of complete DMEM for 48 h. Following co-culture, non-adherent CD4^+^T cells were collected by aspirating the supernatant, and were subjected to qPCR analysis of the expressions of T-bet, IL-4, and IL-17A.

### 4.14. Enzyme-Linked Immunosorbent Assay (ELISA)

Spleen single-cell suspensions were seeded in 48-well plate with density of 7.5 × 10^6^ cells/mL. The cells were cultured with ultraviolet-inactive *C. muridarum* (UV-*C. muridarum*, 1 × 10^5^ IFU/mL) and maintained at 37 °C in a 5% CO_2_ incubator for 48 h. Supernatants were obtained after centrifugation. Culture supernatants were measured and their level of IL-4 secretion was determined using ELISA (Solarbio, Beijing, China) as per the manufacturer’s protocol.

### 4.15. Statistical Analysis

GraphPad Prism 9 software was used in performing statistical analyses. One-way ANOVA or two-way ANOVA was used to compare groups. Data are presented as mean ± SD. Significance level of *p*-value was taken at *p* < 0.05 and it was considered statistically significant (* *p* < 0.05; ** *p* < 0.01; *** *p* < 0.001; **** *p* < 0.0001).

## 5. Conclusions

HDAC6 exerts a protective immunoregulatory function by maintaining immune homeostasis, specifically through suppressing both Th2 cell differentiation and M2 macrophage polarization during *C. muridarum* infection. HDAC6 further mediates the maintenance of this immune balance; its deficiency disrupts such balance and in turn drives Th2-M2 axis dysregulation, which exacerbates immunopathological damage at the infection site and impairs the host’s pathogen clearance ability. These findings identify HDAC6 as a potential therapeutic target for infections and inflammatory diseases characterized by immune imbalance.

## Figures and Tables

**Table 1:** qPCR primer sequences.

Gene Name	Forward Sequence (5′-3′)	Reverse Sequence (5′-3′)
β-Actin	GGCTGTATTCCCCTCCATCG	CCAGTTGGTAACAATGCCATGT
HDAC6	TCCACCGGCCAAGATTCTTC	GCCTTTCTTCTTTACCTCCGCT
16S rRNA	CGCCTGAGGAGTACACTCGC	CCAACACCTCACGGCACGAG
tnfα	CTGAACTTCGGGGTGATCGG	GGCTTGTCACTCGAATTTTGAGA
il-1β	GAAATGCCACCTTTTGACAGTG	TGGATGCTCTCATCAGGACAG
stat6	TGGAGAGCATCTATCAGAGGGA	GCGG AACTCTTCTATAACAGCTT
gata3	AAGCTCAGTATCCGCTGACG	GTTTCCGTAGTAGGACGGGAC
il-4	GGTCTCAACCCCCAGCTAGT	GCCGATGATCTCTCTCAAGTGAT
il-10	CTTACTGACTGGCATGAGGATCA	GCAGCTCTAGGAGCATGTGG
il-13	GCAGCATGGTATGGAGTG	GGTCCTGTAGATGGCATTG
TGF-B	AAAACAGGGGCAGTTACTACAAC	TGGCAGATATAGACCATCAGCA
stat4	GCACTCAGTAAGATGACGCAG	CCAGTAGGGTAAAGCAGTTCTG
T-bet	AACCGCTTATATGTCCACCCA	CTTGTTGTTGGTGAGCTTTAGC
il-12p40	TGGTTTGCCATCGTTTTGCTG	ACAGGTGAGGTTCACTGTTTCT
il-12p35	CAATCACGCTACCTCCTCTTTT	CAGCAGTGCAGGAATAATGTTTC
stat3	CACCTTGGATTGAGAGTCAAGAC	AGGAATCGGCTATATTGCTGGT
RORγt	GAGATGCTGTCAAGTTTGGC	TGTAAGTGTGTCTGCTCCGC
il-22	ATGAGTTTTTCCCTTATGGGGAC	GCTGGAAGTTGGACACCTCAA
il-23	CAGCAGCTCTCTCGGAATCTC	TGGATAC GGGGCACATTATTTTT
ccl2	CTCATAGCAGCCACCTTCATTC	CAAGTCTTCGGAGTTTGGGTTT
ccl3	GGCTGTATTCCCCTCCATCG	CCAGTTGGTAACAATGCCATGT
ccl4	TCCACACAATGGTTTATCAACGG	CACTGTCCTGAAAACTGGCCT
iNOS	ACATCGACCCGTCCACAGTAT	CAGAGGGGTAGGCTTGTCTC
Arg-1	TTGGGTGGATGCTCACACTG	GTACACGATGTCTTTGGCAGA

## Data Availability

The original contributions presented in this study are included in the article/Supplementary Materials. Further inquiries can be directed to the corresponding author.

## Supplementary Materials

The following supporting information can be downloaded at: https://www.mdpi.com/article/10.3390/ijms27073009/s1.
